# Receptors and Lethal Effect of *Bacillus thuringiensis* Insecticidal Crystal Proteins to the *Anticarsia gemmatalis* (Lepidoptera, Noctuidae)

**DOI:** 10.1155/2013/940284

**Published:** 2013-09-30

**Authors:** Lidia Mariana Fiuza, Neiva Knaak, Rogério Fernando Pires da Silva, João Antônio Pêgas Henriques

**Affiliations:** ^1^UNISINOS, Área 2, Laboratório de Microbiologia e Toxicologia, Avenida Unisinos 950, 93022 São Leopoldo, RS, Brazil; ^2^UFRGS, Fitossanidade, Porto Alegre, RS, Brazil; ^3^UFRGS, Biotecnologia, Porto Alegre, RS, Brazil

## Abstract

Bioassays with insecticidal crystal proteins (ICPs) from *Bacillus thuringiensis* have demonstrated that Cry1Aa, Cry1Ac, and Cry1Ba are the most active toxins on larvae of the *Anticarsia gemmatalis*. The toxins Cry1Da and Cry1Ea are less toxic, and toxins Cry2Aa are not active. Binding of these ICPs to midgut sections of the *A. gemmatalis* larvae was studied using streptavidin-mediated detection. The observed staining patterns showed that Cry1Aa and Cry1Ac bound to the brush border throughout the whole length of the midgut. However, the binding sites of Cry1Ba were not evenly distributed in the midgut microvilli. The *in vivo* assays against larvae of 2nd instar *A. gemmatalis* confirmed the results from the *in vitro* binding studies. These binding data correspond well with the bioassay results, demonstrating a correlation between receptors binding and toxicity of the tested ICPs in this insect.

## 1. Introduction

*Bacillus thuringiensis* is one of the most widely used microorganisms for the biological control of insects [1–3]. This gram-positive spore-forming bacterium characteristically produces crystals containing one or several insecticidal crystal proteins—ICPs [4–7]. A novel nomenclature has been proposed based exclusively on amino acid identity. Currently, more than 560 *cry *genes have been identified and classified into 68 classes based on the homology of their proteins (http://www.lifesci.sussex.ac.uk/home/Neil_Crickmore/Bt). The cry genes code for proteins with a range of molecular masses from 50 to 140 kDa [8, 9]. Lepidopteran-specific ICPs are typically produced as bipyramidal crystals that are solubilized in the often alkaline environment (pH 10 to 12) of the larval midgut [10–13]. These proteins, belonging to the Cry1 class of ICPs, are protoxins with a range of molecular masses from 130 to 140 kDa [9, 14] that are proteolytically activated by midgut proteases to toxic protease-resistant fragments (55 to 70 kDa) corresponding to the N-terminal half of the protoxin [14–16]. The delta-endotoxins bind with high affinity to proteins located in the midgut brush border membrane of susceptible insects [17–21]. Following the binding, the toxic fragment or a part of it inserts in to the membrane forming pores [22–24]. The formation of pores in the plasmatic membrane of the cells causes an ionic unbalance between the cytoplasm and the outside environment of the cell. The first effects are the stoppage of feeding and the paralysis of the gut, which causes the insect to die [5, 14, 23, 24].

Traditionally, the binding has been studied using native or biotinylated ICPs and midgut tissue sections [25, 26]. Using intoxicated insects, tissue sections can also be used to examine the histological effects of ICPs and their localization within the gut tract. Gross histological changes such as the enlargement of epithelial cells, the vacuolization of the cytoplasm, the hypertrophy or lyses of cells, and the disruption of the microvilli have been observed in the midgut using this technique [26–28]. Another type of binding site analysis using ligand blot demonstrated that the Cry1Ac toxin binds to a 120 kDa protein in *Manduca sexta* [22, 29]. This 120 kDa protein was identified as aminopeptidase N [30]. Different insect proteins have been identified as receptors for cry proteins the 120 kDa aminopeptidase N Cry1Ac toxin-binding protein purified from brush border vesicles of *Manduca sexta*, *Heliothis virescens*, and *Lymantria dispar* [30–34]. The Cry1Aa toxin binds a 120 kDa protein-like aminopeptidase N [35] the 210 kDa cadherin-like glycoprotein Cry1Ab toxin-binding protein purified from membranes of *Manduca sexta* and *Ostrinia nubilalis* [36, 37].

Receptor binding has been demonstrated to be a key factor in the specificity of ICPs. Indeed, positive correlation between the toxicity and the binding to the brush border membrane [19, 21, 25, 38, 39] has been found in many cases, although this correlation is not necessarily quantitative. An inverse correlation between the binding affinity and toxicity has been reported for *Lymantria dispar* [40]. It can be concluded that the binding is necessary but not sufficient for the toxicity. Specific binding involves two steps, one that is reversible and one that is irreversible. Other data suggest that toxicity correlates with irreversible binding [41]. Irreversible binding might be related to the insertion of the toxin into the membrane but could also reflect a tighter interaction of the toxin with the receptor [8]. For one species, different ICP types may bind to the same or to distinct receptors [18, 20, 26, 42–44].

The *Anticarsia gemmatalis *Hübner 1818 (Lepidoptera, Noctuidae) is one of the most important insect pests of soybean [45–47]. *A. gemmatalis*, also known as velvetbean caterpillar, attacks the plants hampering their development and thus causes losses in grain production [47, 48]. The control of this pest can occur naturally under favorable conditions through the presence of natural enemies. When this does not happen, the use of insecticides with high applications per crop is designed to avoid loss in yield [49]. Currently, many adverse factors interfere in the velvetbean caterpillar control, such as the environmental impact that pesticides cause to other unrelated species and even to its natural enemies. The absence of data on the specificity of ICPs in *A. gemmatalis* enabled the realization of this paper, which analyzed the *in vivo *toxicity of different ICPs for *A. gemmatalis *and the presence of *in vitro* ICPs receptors in the midgut of this insect. The results should contribute to what is known about the toxicity of ICPs in *A. gemmatalis*, becoming an important tool in the management of the target insect.

## 2. Materials and Methods

### 2.1. *B. thuringiensis* ICPs

The cry proteins were obtained from a variety of sources including Pasteur Institut (IEBC, Paris, France) and Pant Genetics Systems (PGS, Ghent, Belgium): Cry1Aa: *B. thuringiensis dendrolimus* HD37 or from *B. thuringiensis thuringiensis *recombinant strain 407 (pHT408) (Kind gift of Dr. M.-M. Lecadet, Institut Pasteur, Paris, France); Cry1Ac: *B. thuringiensis kurstaki* HD73; Cry1Ba: *B. thuringiensis thuringiensis* 4412; Cry1Da: *B. thuringiensis aizawai* HD68; Cry1Ea: *B. thuringiensis darmstadiensis* HD146, and Cry2Aa: was produced by a recombinant *B. thuringiensis *strain expressing the *cry2Aa *gene from *B. thuringiensis kurstaki *HD-1.

**Figure 1:**
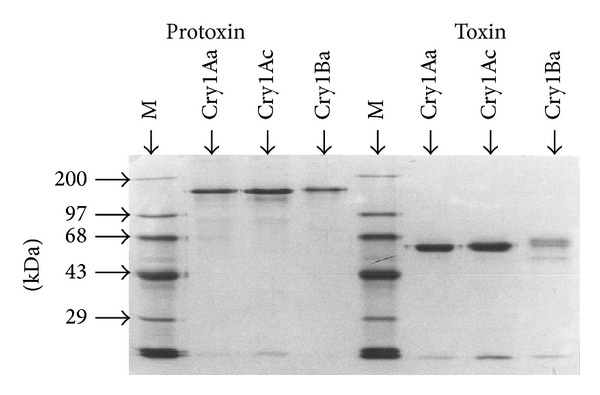
10% SDS polyacrylamide gel electrophoresis *B. thuringiensis* ICPs: Cry1Aa, Cry1Ac, and Cry1Ba protoxins and toxins (equal volumes (2.5 *μ*L) of protein activated (toxin) by incubation with bovine pancreatic trypsin (Type I; Sigma)), molecular mass markers (M).

The *B. thuringiensis* strains were grown as described by Mahillon and Delcour [50]. The autolyzed culture was centrifuged and washed in a phosphate buffer (100 mM NaH_2_PO_4_, 100 mM NaCl, and 0.01% Triton X-100; pH 6). Crystals were separated from spores and debris using saccharose gradients (67 to 88% w/v). The bands containing pure crystals were extensively washed and resuspended in distilled water containing 0.1 mM phenylmethylsulfonyl (PMSF) and stored at −20°C. Crystal proteins were dissolved by incubation for 1 h at 37°C in an alkaline buffer (50 mM Na_2_CO_3_, 10 mM dithiothreitol, and 0.1 mM PMSF; pH 10). The pH of the solution containing protoxins was adjusted to 8.6 by extensive dialysis against 20 mM Tris, and the protoxins were activated by incubation with bovine pancreatic trypsin (Type I; Sigma) (1 *μ*g of per 20 *μ*g of protein) for 2 h. The trypsin of the solution containing *δ*-endotoxins was inactivated by adding 0.5 mg trypsin inhibitor (Type II-S; Sigma) per mg of trypsin. The purity and integrity of delta-endotoxins samples were checked on a 10% sodium dodecyl sulfate-polyacrylamide gel [51] stained with Coomassie blue, according Figure 1. Protein concentrations were determined according to the method of Bradford [52] using bovine serum albumin (BSA) as a standard.

### 2.2. Insects and Bioassays

*A. gemmatalis* larvae were collected from soybean fields in Southern Brazil. The insects were maintained in the laboratory at 25 ± 2°C, 12 hours photoperiod, and 70% relative humidity (RH), and the larvae were reared on an artificial diet described by Greene et al. [53]. The bioassays were performed on second instar larvae (L_2_). The first step was established in a pilot test using 3·10^7^ cells/mL by each strain. Five concentrations of the trypsin-activated *B. thuringiensis* ICPs (Cry1Aa, Cry1Ac, Cry1Ba, Cry1Da, Cry1Ea, and Cry2Aa) were prepared in a phosphate-saline buffer (PBS: 10 mM K_2_HPO_4_, 150 mM NaCl; pH 7.4). Aliquots (100 *μ*L) were applied to the surface of the artificial diet in 9.6 cm^2^ petri dishes, and the larvae were placed in each petri dish. Three repeats per bioassay were performed using fifty larvae for each toxin concentration. In controls, the toxins were replaced by 100 *μ*L of PBS. Mortality was recorded after 7 days, and the results were analyzed by Probit analysis using Polo-PC Program LeOra Software, 1987 [54].

### 2.3. Biotinylation of ICPs

Activated ICPs were biotinylated according to the procedure [55, 56]. 40 *μ*L of biotinyl hydroxysuccinimide ester (Amersham) was added to 1 mg of toxin dissolved in a sodium bicarbonate buffer (100 mM NaHCO_3_, 150 mM NaCl; pH 9). Following a 1 h of incubation at room temperature, the reaction mixture was applied to a Sephadex G-25 column (Sigma) in order to separate the biotinylated toxins from free biotin. The toxin was eluted with sodium bicarbonate buffer. The fractions containing biotinylated ICPs were identified by dot blot analysis. 1 *μ*L of fraction was spotted onto a nitrocellulose membrane and incubated with streptavidin-alkaline phosphatase conjugate (diluted 1/300 in Tris-saline-Triton buffer (TST: 10 mM Tris, 150 mM NaCl, and 0.1% Triton X-100; pH 7.6)) for 1 h. Biotinylated delta-endotoxins was visualized by incubation with an alkaline phosphatase substrate solution (1.75 mg of 5-bromo-4-chloro-3-indolyl phosphate and 2.5 mg of nitroblue tetrazolium in 10 mL of buffer containing 100 mM Tris, 100 mM NaCl, and 5 mM MgCl_2_; pH 9.5). The concentration of biotinylated delta-endotoxins was measured as described by Bradford [52] using BSA as a standard. The purity and integrity of biotinylated delta-endotoxins were checked by SDS-PAGE analysis followed by electroblotting onto a nitrocellulose membrane (Sigma) in a 0.5x Towbin buffer (12.5 mM Tris, 96 mM glycine; pH 8.3 with 10% methanol). Blotted membranes were developed using the same technique for dot-blot.

### 2.4. Histological Sections

Midguts of fifth instar larvae (L_5_) of *A. gemmatalis* were dissected and fixed in Bouin Hollande 10% sublimate [57] for 24 h, washed for 12 h in distilled water, and dehydrated with a series of ethanol baths (once at 70% ethanol, twice at 96% ethanol, and twice at 100% ethanol for 1 h each). The tissues were then infiltrated in mixed-baths (50% ethanol : 50% toluol, 50% toluol : 50% paraplast) and twice impregnated with 100% paraplast before being embedded at 58°C. Finally, the paraplast was hardened at 4°C [27, 56]. Longitudinal sections, 7 *μ*m thick, were cut with a microtome and were placed on mounting glasses previously coated with a 10% poly-l-lysine section (Sigma).

### 2.5. *In Vitro* Binding of ICPs on Tissue Sections

*In vitro* detection of toxin binding was studied on tissue sections from isolated guts of untreated larvae. Tissue sections were deparaffinized and dehydrated with successive incubations: twice for 5 min with 100% toluol, three times for 3 min with 100% ethanol. The sections were washed with distilled H_2_O for 1 min, treated with lugol (0.5% I_2_ in H_2_O), in order to remove HgCl_2_ (Aldrich), and subsequently immersed for 2 min in a 5% Na_2_S_2_O_5_ solution. After washing the tissues with distilled water for 1 min, they were equilibrated in TST buffer for 5 min. Prior to the incubation with toxin, the sections were treated with a blocking solution (1% blocking reagent (Boehringer) in a TST buffer) in order to inhibit nonspecific binding.

The tissue sections were then incubated with 1.5 to 6 *μ*g of biotinylated toxins (Cry1Aa, Cry1Ac, Cry1Ba, Cry1Da, Cry1Ea, and Cry2Aa) in the TST-buffer for 1 h. Following a washing step with the TST-buffer, the tissues were covered with 300 *μ*L of streptavidin-alkaline phosphatase conjugate (Amersham) diluted 1/300 in TST-buffer. Following a 1 h incubation period, unbound streptavidin-enzyme conjugate was removed by washing with the TST-buffer and the bound toxin was finally visualized by incubation with an alkaline phosphatase substrate solution (1.75 mg of 5-bromo-4-chloro-3-indolyl phosphate and 2.5 mg of nitroblue tetrazolium in 10 mL of buffer containing 100 mM Tris, 100 mM NaCl, and 5 mM MgCl_2_; pH 9.5) or a peroxidase substrate (0.01% 3,34-diaminobenzidine, 0.003% H_2_O_2_ in 50 mM Tris; pH 7.6). The reaction was stopped by transferring the samples to the TST buffer. Finally, in order to preserve the stained sections, the tissues were dehydrated and mounted with Clearium medium.

Negative controls were performed by omission of toxins or biotinylated toxins or enzyme-conjugated streptavidin. Staining was not observed when individual steps were omitted.

## 3. Results

### 3.1. Toxicity of ICPs on *A. gemmatalis* Larvae

The delta-endotoxins tested purity and integrity were evaluated in SDS-PAGE according to Figure 1. The data of the bioassays showed that five ICPs were toxic against larvae of 2nd instar *A. gemmatalis*. In these bioassays, after 48 hours of treatments (3·10^7^ cells/mL), the strains *B. thuringiensis dendrolimus* HD37 (and from *B. thuringiensis thuringiensis *recombinant strain 407-pHT408), *B. thuringiensis kurstaki* HD73, *B. thuringiensis thuringiensis *4412, *B. thuringiensis aizawai* HD68, and *B. thuringiensis darmstadiensis* HD146 caused 80% mortality corrected. However, the Cry2Aa strain produced by a recombinant *B. thuringiensis *strain expressing the *cry2Aa *gene from *B. thuringiensis kurstaki *HD-1 was not pathogenic against the population of the target species tested when compared to the control (100 *μ*L of PBS).

In assays with purified ICPs, the following results were observed: (i) the strains *B. thuringiensis dendrolimus* HD37 (and from *B. thuringiensis thuringiensis *recombinant strain 407-pHT408), *B. thuringiensis kurstaki* HD73, and *B. thuringiensis thuringiensis *4412 caused 90% mortality race in 48 hours after treatment application; (ii) the strains *B. thuringiensis aizawai* HD68, *B. thuringiensis darmstadiensis* HD146 caused 85% and 65% mortality, respectively, over a period between 144 and 168 hours after application of the proteins; and (iii) the recombinant *B. thuringiensis *strain expressing the *cry2Aa *gene from *B. thuringiensis kurstaki *HD-1 showed no significant mortality compared to the control (100 *μ*L of PBS). 

*In vivo* assays to determine the median lethal concentration (LC_50_) native toxins (ICPs) were used, which was obtained from the purified crystals. In these assays, three ICPs were applied (Cry1Aa: *B. thuringiensis dendrolimus* HD37 or from *B. thuringiensis thuringiensis *recombinant strain 407-pHT408; Cry1Ac: *B. thuringiensis kurstaki* HD73; and Cry1Ba: *B. thuringiensis thuringiensis* 4412) which caused the greatest acute toxicity in preliminary tests. The LC_50_ of native toxins Cry1Aa, Cry1Ac, and Cry1Ba was less than 0.1 *μ*g/larvae on the seventh day after the treatment when compared to the corresponding positive control (strain *B. thuringiensis kurstaki* HD1) whose LC_50_ equivalent to 0.082 *μ*g/larvae (0.056–0.105) on the fifth day after the treatment.

### 3.2. ICPs Receptors Binding Sites to *A. gemmatalis* Larvae Tissue Sections

The three biotinylated toxins (Cry1Aa, Cry1Ac, and Cry1Ba) with greater lethal effect on larvae of 2nd instar *A. gemmatalis* were studied on midgut tissue sections from untreated larvae. Bound biotinylated ICP was visualized using and streptavidin-mediated detection techniques.

**Figure 2:**
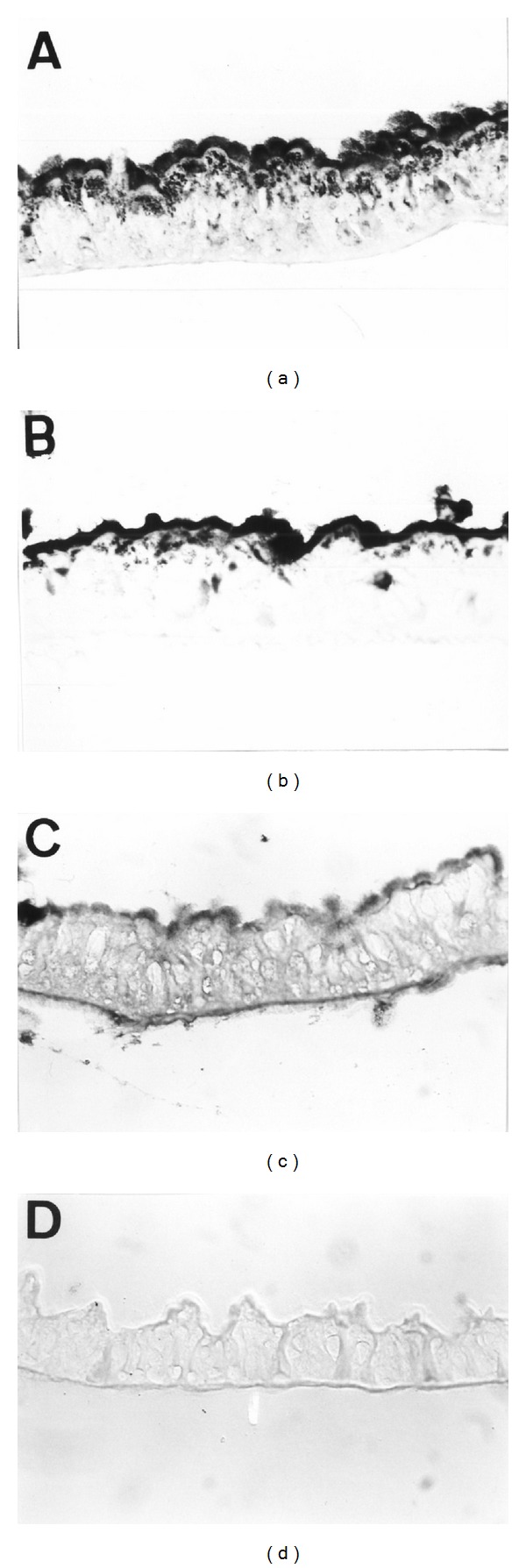
Detection of *in vitro* bound biotinylated ICPs on *A. gemmatalis* guts tissue: Cry1Aa (a), Cry1Ac (b), and Cry1Ba (c). Negative control (d) when tissue sections were incubated with the biotinylated ICPs (e.g., Cry1Aa) and omission of AP-conjugated streptavidin or when tissue section were incubated with the AP-conjugated streptavidin and omission of biotinylated ICPs. Light micrograph obtained with Nomarski differential interference contrast illumination.

Incubation with Cry1Aa (Figure 2(a)) and Cry1Ac (Figure 2(b)) resulted in very intense staining of the brush border along the whole length of the midgut. Staining due to binding of Cry1Ba (Figure 2(c)) was also intense but not evenly distributed throughout the midgut. No labeling was observed with control samples to the brush border membrane (Figure 2(d)).

## 4. Discussion

The *Cry1* gene of *B. thuringiensis* ICPs were a great promise for the control of lepidopteran pests on soybean, either as a microbial insecticide or by being genetically engineering into the soybean plant [58]. In this study, we have analysed toxicity and binding of six ICPs to larvae of *A. *Bioassays using trypsin-activated ICPs, and second instar of *A. gemmatalis* indicated that among the six proteins tested, Cry1Aa, Cry1Ac, and Cry1Ba were most active on this insect. Cry1Da and Cry1Ea were less active while Cry2Aa was essentially not active. 

Also in search of biological control of the velvetbean caterpillar, the pathogenicity of twelve *B. thuringiensis* isolates was tested against *A. gemmatalis* [59]. Those authors performed a series of bioassays by feeding third instar larvae of* A. gemmatalis *with artificial diets containing the *B. thuringiensis* spore-crystal complex (3·10^8^ cells/mL), where four new isolates (U87-2, U98-1, U98-4, and IP01) showed that larval mortality of *A. gemmatalis* similar or greater as standard strain (*B. thuringiensis kurstaki *HD1—Dipel), and the PCR technique was used to amplify DNA fragments related to the known *cry1* genes. The toxic* B. thuringiensis* isolates also exhibited an expected protein profile when total protein extracts were evaluated by SDS-PAGE [59], confirming that the Cry1 toxins are toxic potential for the species studied in our work (velvetbean caterpillar).

However, another new *B. thuringiensis* (*Bt117-4*) isolate that amplifies fragments corresponding to *cry2 *and *cry9 *genes, which synthesize protein fragments of equivalent to 130, 90, and 45 kDa [58]. The transmission electron microscopy revealed the presence of protein crystals and the CL_50_ with Cry-purified proteins corresponded to 0.195 *μ*g/larvae of the second instar of *A. gemmatalis, *whose data were very similar to this paper.

In order to study the interaction of ICPs with the digestive tract of *A. gemmatalis*, we have studied *in vitro* receptors binding of biotinylated ICPs to midgut tissue sections of healthy larvae. In this way, these results generally correlate with levels of toxicity of the different ICPs. For instance, the most toxic proteins, Cry1Aa and Cry1Ac, bind strongly *in vitro* and result in strong detection staining signals. In contrast, Cry1Ba was less active than the above mentioned Cry1 ICPs, displaying less intense *in vitro* binding to tissue sections, which render the affinity for this toxin even lower than in the insect midgut. The results from our receptors binding studies on tissue slides are in agreement with the observations from binding experiments with radiolabeled toxins on brush border membrane vesicles from *C. suppressalis* [18, 20]. Fiuza et al. [18] demonstrated that both Cry1Aa and Cry1Ac bind with high affinity whereas Cry1Ba binds with somewhat lower affinity.

On the basis of the general correlation between toxicity and receptors binding to the brush border observed in this study, it may be suggested to use these *in vitro* binding detection methods as a means of screening in order to quickly select *B. thuringiensis* ICPs which could have an *in vivo* effect on Lepidoptera larva and other insect species, as indicated by Denolf et al. [26, 56]. These methods, however, are obviously of a qualitative rather than a quantitative nature, since among ICPs with significant insecticidal activity there is no clear correlation between staining intensity and toxicity. This lack of a quantitative correlation has been observed previously for *O. nubilalis* [26]. Likewise, in many cases, there is no quantitative correlation between binding parameters such as binding affinity or receptor site concentration and toxicity. Clearly, if screening by binding is performed, the ICPs that show binding to tissue of the target insect need to be tested in bioassays to confirm biological activity.

## 5. Conclusion

The present study shows that *B. thuringiensis* ICPs which bind to receptor sites in the midgut of the target insect should be evaluated *in vivo* to estimate the lethal effect, but the toxins that do not have receptors in the guts need not be tested in bioassays. In order to control *A. gemmatalis* larvae, it is possible to use commercial *B. thuringiensis* products containing strains producing ICPs toxic against this species. A helpful alternative would be to express *B. thuringiensis* genes encoding ICPs active against *A. gemmatalis* in soybean plants in order to obtain plants that are resistant to this pest. Currently, several studies confirmed the successful use of soy Bt, for example, Stewart et al. [60], these authors used somatic embryos of Jack, a *Glycine max* Merrill cultivar, were transformed using microprojectile bombardment with a synthetic *Bacillus thuringiensis* insecticidal crystal protein gene (crylAc) driven by the 35s promoter and linked to the HPH gene. In detached-leaf bioassays, plants with an intact copy of the Bt gene, and to a lesser extent those with the rearranged copy, were protected from damage from *Helicoverpa zea*, *Pseudoplusia includens*, *Heliothis virescens*, and *Anticarsia gemmatalis*. Corn earworm produced less than 3% defoliation on transgenic plants compared with 20% on the lepidopteran-resistant breeding line CatlR81-296 and more than 40% on susceptible cultivars. According to our results, it would be advisable to use ICPs Cry1Aa, Cry1Ac, or Cry1Ba.
